# Hematopathological Patterns in Acute Myeloid Leukemia with Complications of Overt Disseminated Intravascular Coagulation

**DOI:** 10.3390/diagnostics15030383

**Published:** 2025-02-06

**Authors:** Bernhard Strasser, Sebastian Mustafa, Josef Seier, Josef Tomasits, Alexander Haushofer

**Affiliations:** 1 Institute of Clinical Chemistry and Laboratory Medicine, Klinikum Wels-Grieskirchen 1, 4600 Wels, Austria; josef.tomasists@kepleruniklinikum.at (S.M.); alexander.haushofer@klinikum-wegr.at (A.H.); 2 Department of Internal Medicine, Johannes Kepler University, 4040 Linz, Austria; josef.tomasits@kepleruniklinikum.at; 3 Medical University Graz, 8036 Graz, Austria

**Keywords:** acute myeloid leukemia, AML, disseminated intravascular coagulation, DIC, cup-like-blasts, hematopathology, molecular diagnostics, *FLT3* mutations, *NPM1* mutations, acute promyelocytic leukemia

## Abstract

**Background:** Acute myeloid leukemia (AML) complicated by disseminated intravascular coagulation (DIC) poses major diagnostic and therapeutic challenges. While DIC is well documented in acute promyelocytic leukemia, its manifestations in non-APL AML remain underexplored, necessitating precise diagnostic strategies for effective management. **Methods:** AML patients with overt DIC were analyzed, including morphological, immunophenotypic, cytogenetic, and genetic evaluations. DIC was diagnosed using the ISTH scoring system, and AML subtypes were classified following WHO criteria. **Results:** Three diagnostic patterns were identified. (1) Acute promyelocytic leukemia: Leukemia characterized by *PML::RARa* rearrangements, *FLT3* co-mutations, and frequent Auer rods and faggot bundles. Immunocytological analysis showed CD34 and HLA-DR negativity. (2) AML with *FLT3* and/or *NPM1* mutations: A high prevalence of cup-like blasts was found in 70% of cases. *FLT3* mutations, often co-occurring with *NPM1*, dominated, while karyotypes were typically normal. Immunophenotyping revealed strong myeloid marker expression (MPO+, CD13+, and CD33+), with occasional CD34 negativity. (3) AML with monocytic differentiation: Leukemia defined by monoblastic/promonocytic morphology, *DNMT3A* mutations, and complex karyotypes or 11q23 rearrangements. Immunophenotyping demonstrated a dominance of monocytic markers (CD4+, CD14+, CD15+, and CD64+). Two patients presented unique profiles with no alignment to these patterns. **Conclusions:** This study highlights distinct hematopathological patterns of AML with overt DIC, providing a framework for early and precise diagnosis. Recognizing these patterns is critical for tailoring diagnostic and therapeutic approaches to improve outcomes in this high-risk population.

## 1. Introduction

Disseminated intravascular coagulation (DIC) is a serious complication of acute myeloid leukemia (AML) characterized by the systemic activation of coagulation pathways, leading to microvascular thrombi, the consumption of clotting factors, and life-threatening bleeding. [1]. The International Society on Thrombosis and Haemostasis (ISTH) has established a scoring system for diagnosing overt DIC based on laboratory parameters such as platelet count, prothrombin time (PT), fibrinogen, and fibrin-related markers [2]. Moreover, revisions to the cut-off values for those fibrin-related markers, such as D-dimer, have led to the development of an updated scoring system to enhance diagnostic accuracy and reduce the risk of overdiagnosis [3]. A score of ≥4 (Table 1) points is indicative of overt DIC, which is associated with high mortality and adverse clinical outcomes in AML patients [3,4].

In clinical practice, the urgency of diagnosing AML early is underscored by its potential life-threatening complications, such as neutropenic infections or coagulopathies. While acute promyelocytic leukemia (APL) is a well-known subtype with a high risk of DIC due to *PML::RARa* rearrangements, recent studies have highlighted that non-APL AML also frequently presents with DIC. This necessitates a broader diagnostic framework to identify at-risk patients early [5,6].

Modern treatment strategies for AML have shifted towards a paradigm of personalized medicine, with an increasing emphasis on genetic and molecular profiling to guide therapy. This approach allows for the use of targeted treatments, such as gemtuzumab ozogamicin for core-binding factor AML, midostaurin for *FLT3*-mutated AML, CPX-351 for AML with myelodysplasia-related changes, and ivosidenib for *IDH1*-mutated AML. Recent studies suggest that in clinically stable AML patients, delaying the initiation of induction therapy to allow for comprehensive genetic testing does not negatively impact survival and may provide considerable benefits by enabling individualized treatment. However, in critical situations, such as patients presenting with life-threatening complications, including DIC, delaying treatment is not feasible. In these cases, the rapid initiation of therapy, including cytoreduction and an aggressive AML-specific treatment regimen, is essential to stabilize the patient and prevent fatal outcomes [7,8,9].

Previous research on DIC in AML has predominantly focused on therapeutic options and prognostic factors associated with this severe condition [10,11]. However, the early identification of patients at risk for DIC complications is critical, as it directly influences treatment decisions and overall patient management. Consequently, there is a pressing need for diagnostic studies aimed at identifying reliable indicators of DIC in AML. Hematopathologists are central to this diagnostic process, as they perform microscopic, immunophenotypic, and genetic evaluations to confirm AML and identify its subtypes [12,13]. This study aims to elucidate the hematopathological patterns of AML complicated by overt DIC, providing insights into the diagnostic challenges and implications for patient management.

## 2. Materials and Methods

**Figure 1:**
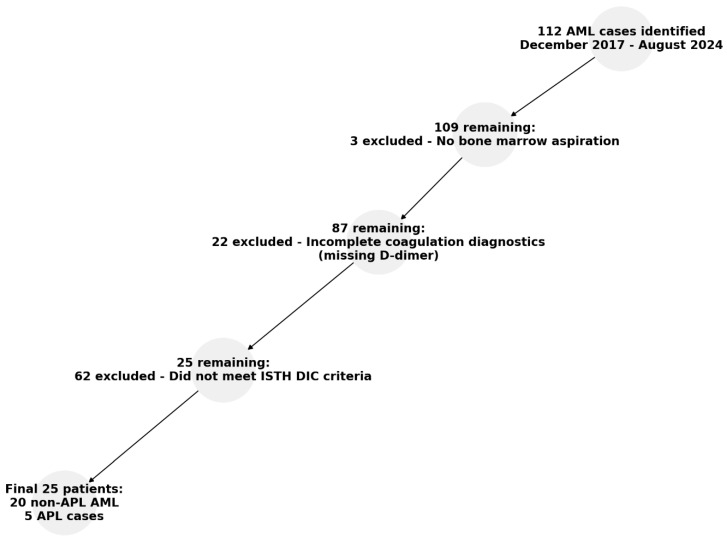
Overview of patient selection for the study. The flowchart illustrates the recruitment process, starting with 112 identified AML cases and applying exclusion criteria step-by-step for a final 25 patients included in the study.

This study was conducted as a retrospective observational analysis of patients diagnosed with AML complicated by overt DIC. A total of 112 AML cases were identified from the institutional database of the Klinikum Wels-Grieskirchen between December 2017 and August 2024. Inclusion was based on a confirmed AML diagnosis according to the World Health Organization (WHO) classification (5th edition) and an ISTH overt DIC score of ≥4 at the time of presentation. Patients with incomplete clinical or laboratory data were excluded from the analysis. Comprehensive clinical, laboratory, and pathological data were collected for each patient. Key parameters included platelet count, prothrombin time (PT), fibrinogen levels, and D-dimer levels, which were assessed as part of the ISTH-DIC scoring system. The patient selection process for the study was conducted stepwise, as illustrated in Figure 1. In the first step, 3 patients were excluded because no bone marrow aspiration was performed. In the second step, 22 patients were excluded due to incomplete coagulation diagnostics, particularly the absence of D-dimer measurements. In the final step, the ISTH overt DIC criteria were applied, leaving 25 patients eligible for inclusion in the study. These included 20 non-APL AML cases and 5 APL cases. Additional data on clinical presentation of overt DIC related complications were obtained from medical records. Morphological evaluation of bone marrow aspirates was performed by experienced hematopathologists. For this study, archived bone marrow aspirates from all included cases were reevaluated microscopically to confirm findings and identify specific features, such as myeloblast morphologies. Immunophenotyping was conducted using flow cytometry to assess expression levels of markers including CD34, HLA-DR, MPO, CD13, CD33, CD14, and others as appropriate for AML subtyping. Flow cytometric analysis was performed using a BD FACS Lyric^TM^ flow cytometer (BD Biosciences, San Jose, CA, USA). The panel of fluorophore-conjugated antibodies and gating strategy is described in detail in the Supplementary Data. Bone marrow samples were processed into single-cell suspensions and stained with fluorophore-conjugated antibodies targeting AML-relevant markers. Compensation controls were prepared to correct for spectral overlap. Samples were acquired using a BD FACS Lyric cytometer. Acquisition settings were optimized by adjusting forward and side scatter (FSC/SSC) to visualize cell populations, and 50,000–100,000 events were collected per sample. Data analysis followed a gating strategy designed for AML. Initial gating excluded debris (FSC vs. SSC) and doublets. Live, non-lymphocytic populations were identified based on CD45 vs. SSC by excluding lymphocytes with high CD45 and low SSC. Myeloblasts were gated in the dim CD45 and low SSC region and confirmed by the expression of CD34 and CD117. Aberrant marker expression (non-myeloid expression on myeloblasts: CD2, CD7, CD10, CD56, TdT) was assessed for further characterization. For CD34-negative or HLA-DR-negative myeloblasts, alternative markers such as CD13, CD33, and CD117 were used to confirm their identity. These populations were evaluated based on co-expression patterns and integrated with clinical and morphological data for comprehensive assessment. A detailed flowchart illustrating the FACS analysis and gating strategy for AML is provided in the Supplementary Materials.

Molecular genetic analysis was carried out using the next-generation sequencing (NGS) Sophia Genetics^®^ (Sophia Genetics SA, Saint-Sulpice, Switzerland) myeloid solution panel (hg19 reference genome), including 30 genes and gene sections of 30 genes that are frequently mutated in AML. This panel provides comprehensive coverage of key genetic alterations, including mutations in *ABL1*, *ASXL1*, *BRAF*, *CALR*, *CBL*, *CEBPA*, *CSF3R*, *DNMT3A*, *ETV6*, *EZH2*, *FLT3*, *HRAS*, *IDH1*, *IDH2*, *JAK2*, *KIT*, *KRAS*, *MPL*, *NPM1*, *NRAS*, *PTPN11*, *RUNX1*, *SETBP1*, *SF3B1*, *SRSF2*, *TET2*, *TP53*, *U2AF1*, *WT1*, and *ZRSR2*. Cytogenetic studies were conducted using G-banding and fluorescence in situ hybridization (FISH) to identify chromosomal abnormalities relevant to AML.

Descriptive statistics were used to summarize patient characteristics and laboratory findings. Comparisons between patient subgroups were performed using the chi-square test or Fisher’s exact test for categorical variables and the Mann–Whitney U test for continuous variables. The study was approved by the Institutional Review Board of Upper Austria/Johannes Kepler University and was conducted in accordance with the 1964 Helsinki Declaration and its later amendments or comparable ethical standards.

## 3. Results

### 3.1. Overt DIC Score Comparison

**Figure 2:**
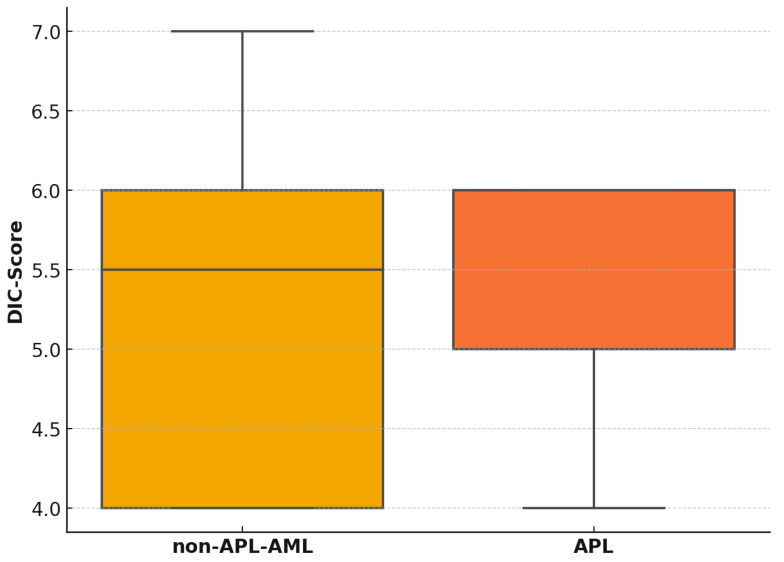
Boxplot illustrating the distribution of DIC scores between the two diagnostic groups, non-APL AML and APL. The median DIC score for both groups appears similar, suggesting no difference in central tendency. The wider IQR observed in the non-APL AML group highlights greater variability in DIC scores. This could reflect the underlying heterogeneity of this diagnostic group, which encompasses a diverse range of molecular subtypes and disease severities.

The mean overt DIC score in non-APL AML patients was 5.25 (median: 5.5); this is comparable to the score in APL patients, who had a mean score of 5.4 (median: 6.0). These results are shown in Figure 2. A total of 25 patients with AML and overt DIC were included in the analysis: 20 with non-APL AML and 5 with APL. Among the individual components of the overt DIC score, the APL patients demonstrated lower platelet counts, fibrinogen levels, and prothrombin times but higher D-dimer levels compared to the non-APL AML patients. These findings indicate a tendency for more pronounced consumption of coagulation factors and platelets in APL cases. In addition to the DIC biomarkers, we also analyzed relevant blood count parameters such as hemoglobin and leukocytes. This showed that hemoglobin was lower in the non-APL AML group than in the APL patients. The analysis of the leukocyte values revealed that six patients had hyperleukocytosis (leukocytes > 100,000/µL) at the time of diagnosis, with five of these patients belonging to the non-APL AML group and one to the APL group. An overview of the overt DIC score parameter is presented in Figure 2 and Table 2.

**Figure 3:**
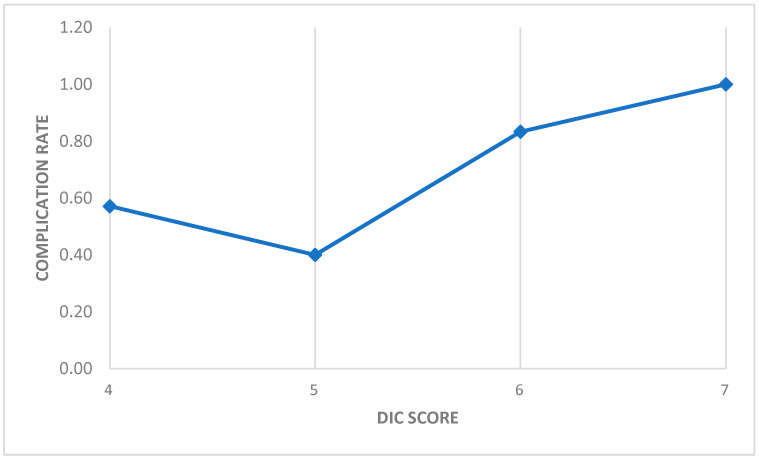
The relationship between DIC scores and complication rates. At a DIC score of 4, 4 out of 7 patients (57%) experienced complications. At a score of 5, 2 out of 5 patients (40%) had complications. For a score of 6, complications were observed in 10 out of 12 patients (83%), and at a score of 7, a single patient in this group experienced complications (100%).

Higher DIC scores showed a tendency towards increased complication rates, with rates ranging from 40% at a DIC score of 5 to 83% at a score of 6, and 100% for a score of 7. These observations suggest a possible association between DIC severity and complications, although the limited number of patients included in the study should be considered. The data are presented in Figure 3.

### 3.2. Key Genetic Drivers and Cellular Characteristics in AML with DIC

We analyzed 25 patients with AML complicated by overt DIC: 5 with APL and 20 with non-APL AML. Across the cohort, 72 mutations were identified, resulting in an average of 2.88 mutations per patient. Patients with non-APL AML demonstrated a higher mutational burden than those with APL (3.1 vs. 2 mutations per patient). The most frequently observed mutations were in *FLT3*, *DNMT3A*, *NPM1*, and *WT1*. Mutations in genes encoding chromatin modifiers (e.g., *ASXL1*) or components of the splicing complex (e.g., *SF3B1*, *SRSF2*, *U2AF1*, and *ZRSR2*), which are commonly associated with AML with myelodysplasia-related changes (AML-MR), were rare. Additionally, no cases of secondary AML arising from prior myelodysplastic syndromes (MDSs) or chronic myelomonocytic leukemia (CMML) were identified in this cohort.

The most prominent mutational pattern in non-APL AML was a triplet mutation involving *DNMT3A*, *FLT3*, and *NPM1*, which was observed in 7 out of 20 patients. Cytogenetic analysis revealed that all APL cases exhibited the pathognomonic t(15;17) translocation, leading to the *PML::RARa* fusion. In contrast, non-APL AML cases were less frequently defined by chromosomal aberrations, with 60% showing a normal karyotype. Specific aberrations observed in non-APL AML included *KMT2A* (11q23) rearrangements, *RUNX1::RUNX1T1* rearrangements, and complex karyotypes. Notably, patients with the *DNMT3A*, *FLT3,* and *NPM1* mutational triplet consistently exhibited a normal karyotype.

**Figure 4:**
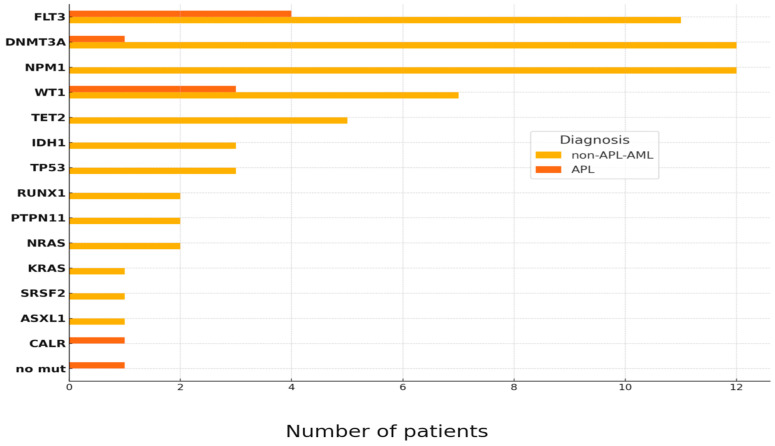

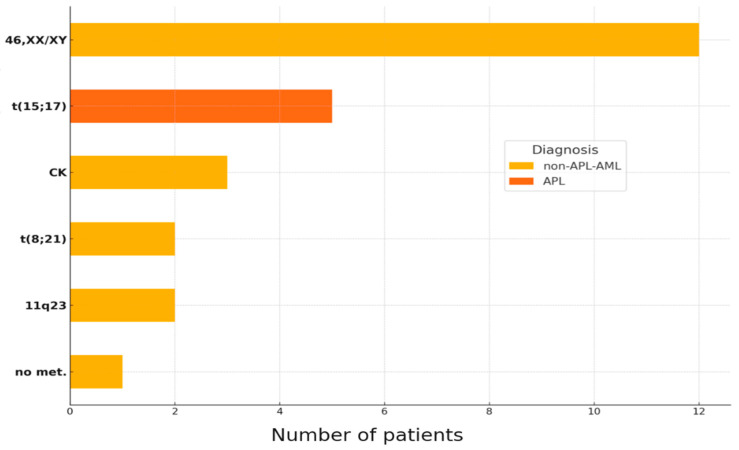
The **upper** graphic depicts the mutational landscape of non-APL AML and APL patients, while the **lower** graphic illustrates the distribution of cytogenetic abnormalities. CK = complex karyotype (≥3 chromosomal abnormalities).

In APL patients, the t(15;17) translocation was strongly associated with FLT3 mutations (four out of five cases). Chromosomal aberrations were consistently present in APL patients (five out of five cases), with the t(15;17) translocation identified as a characteristic feature. In contrast, among 19 evaluable karyograms in non-APL AML (one not evaluable; no metaphase growth), 12 showed normal karyotypes and only 7 revealed aberrations. The mutational burden was lower in APL patients, with an average of 2 mutations per patient compared to 3.1 mutations per patient in non-APL AML. To further evaluate dominant mutational clones in non-APL AML, we defined a mutation as dominant when either (1) it was the sole mutation detected (e.g., *WT1*, *DNMT3A*, or *FLT3* in three patients each) or (2) it displayed a variant allele frequency (VAF) at least 10% higher than other co-occurring mutations. Using this approach, we identified *FLT3* as the dominant clone in six non-APL AML patients, followed by *DNMT3A* and *TP53* in two patients each, and *RUNX1*, *WT1*, and *NPM1* in one patient each. Figure 4 provides an overview of the associated genetic mutations and cytogenetic aberrations. From a cellular perspective, we identified three distinct morphological patterns:

- Patients with promyelocytic neoplastic cells (APL);
- Patients with a dominance of cup-like blasts;
- Patients with predominant monocytic differentiation.

### 3.3. Hematopathological Patterns of AML with Overt DIC

**Figure 5:**
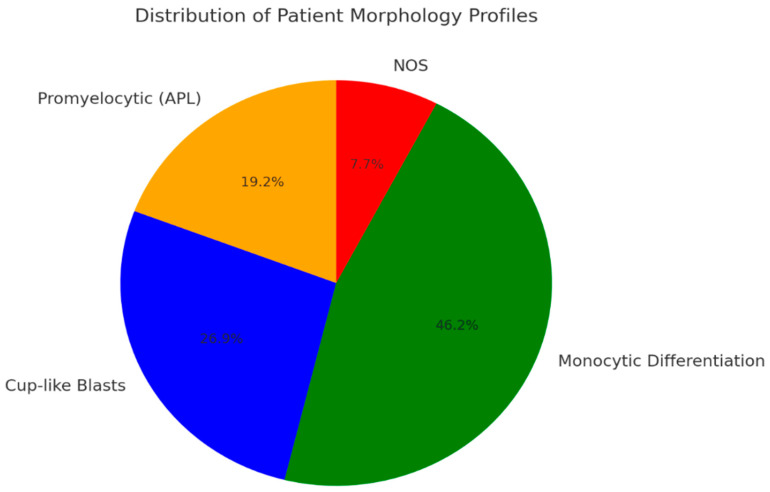
The distribution of patient morphology profiles, categorized into promyelocytic neoplastic cells (APL), cup-like blasts, monocytic differentiation, and Not Otherwise Specified (NOS).

The patient cohort exhibited diverse morphological profiles based on their neoplastic cell types. Among the analyzed cases, 5 patients demonstrated promyelocytic neoplastic cells (APL), 7 had a dominance of cup-like blasts, and 12 showed predominant monocytic differentiation. Additionally, two patients could not be assigned to any of these categories and were classified as “Not Otherwise Specified (NOS)”. An overview of patient groups related to the morphological patterns is given in Figure 5.

#### 3.3.1. APL

APL is among the AML subtypes most strongly associated with overt DIC. During the observational period, we identified seven patients with APL complicated by overt DIC. Two patients were excluded from detailed clinical pathology evaluation due to acute mortality before bone marrow aspiration could be performed. All included patients met the inclusion criterion of detecting the *PML::RARa* fusion. *FLT3* mutations were identified in four out of five patients, with only one patient lacking any additional mutations alongside *PML::RARa.*

**Figure 6:**
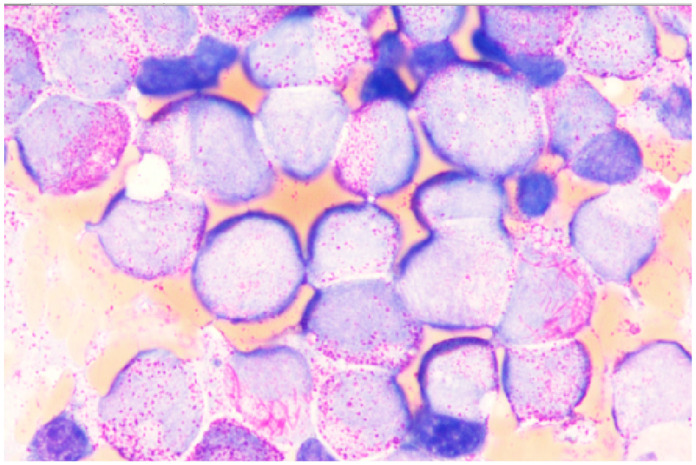
Representative bone marrow smear (100× magnification) showing blast and promyelocyte morphology characteristic of APL with evidence of hypergranulation, Auer rods, and faggot bundles.

Bone marrow examination revealed a high number of neoplastic cells in all APL cases, although differentiating between myeloblasts and promyelocytes was often challenging. Morphological diagnosis of classic APL was facilitated by the uniformity and immaturity of neoplastic cells, with prominent granulations and Auer rods, including the characteristic faggot bundles (Figure 6). In two cases of variant APL (APL-V), bi-nuclear cells were repeatedly observed, prompting *PML::RARa* testing. Immunocytological studies supported the diagnosis, with negativity or weak expression of CD34 and HLA-DR, and aberrant expression of myeloblast markers such as CD22, CD2, or CD19. A summary of APL features is presented in Table 3.

#### 3.3.2. AML with Cup-Like Blast Dominance

In this group, a distinct hemato-oncological phenotype was observed that was characterized by high-grade infiltration of bone marrow by myeloblasts and the presence of *FLT3* and/or *NPM1* mutations. Among the seven patients, *FLT3* co-mutations with *NPM1* were detected in four cases, while two patients had isolated *NPM1* mutations and one patient carried an isolated *FLT3* mutation. A total of 13 mutations were identified (6 *NPM1* and 7 *FLT3*), and all but one patient exhibited normal karyotypes (46,XX or 46,XY); the exception involved a translocation t(8;21).

**Figure 7:**
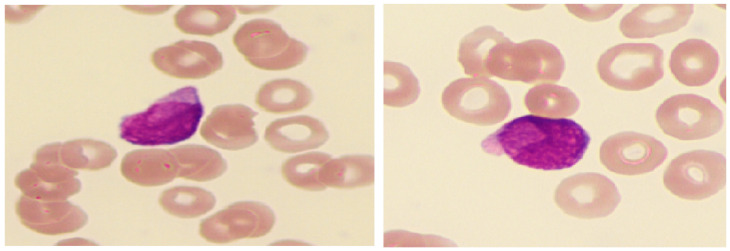
Bone marrow smear showing myeloblasts with cup-like morphology (100× magnification).

Cytomorphological analysis revealed uniform myeloblast morphology across patients, which was characterized by low to abundant cytoplasmic granulation and cases of pseudoblebs or pseudopods. Notably, 70% of patients (5/7) displayed cup-like blasts. The cup-like blasts comprised a small proportion of the myeloblast population in most patients (<10%), with only one patient showing around 10–20% myeloblasts with cup-like morphology. Immunocytological analysis showed a dominance of classical myeloid markers (MPO+, CD13+, and CD33+), with three patients exhibiting a CD34-myeloblast population. Aberrant markers (CD19, CD10, or CD2) were observed in two patients. An overview of hematopathological characteristics is presented in Table 4; typical cup-like blasts are presented in Figure 7.

#### 3.3.3. AML with Monocytic Differentiation

**Figure 8:**
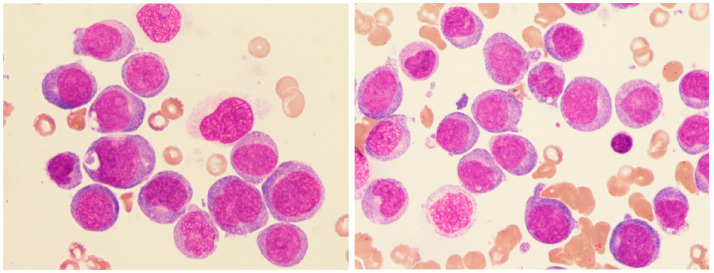
Bone marrow smear from patients with AML showing monoblasts with prominent basophilic cytoplasm, round to oval nuclei, vacuolization, and perinuclear clearing (100× magnification).

We identified 11 patients with AML and overt DIC who exhibited a monocytic differentiation phenotype. Microscopically, these patients were characterized by monoblast infiltration of the bone marrow. Monoblasts were typically enlarged, with round to oval nuclei, a poorly granulated cytoplasm, and dark basophilic cytoplasmic rims. Golgi-like perinuclear clearing and occasional cytoplasmic protrusions were also observed. Furrowed and irregular nuclei were frequently noted, which are indicative of promonocytes (Figure 8).

Morphologically, these cases resembled acute monoblastic leukemia, with some appearing as acute myelomonocytic leukemia. The most frequent mutation was *DNMT3A*, while *FLT3* and *NPM1* mutations did not correlate with cup-like blasts in this group. Cytogenetic abnormalities were found in 5 out of 10 patients, with a tendency toward complex karyotypes and 11q23 rearrangements. Immunophenotyping showed a dominance of monocytic and myelomonocytic markers (CD4+, CD14+, CD15+, and CD64+), with at least two markers expressed in all cases. An overview of hematopathological characteristics is presented in Table 5.

#### 3.3.4. Not Otherwise Specified (NOS) Profile

Two patients did not fit into the three defined phenotypic patterns (APL, AML with cup-like blast dominance, or AML with monocytic differentiation). These patients were classified as having a Not Otherwise Specified (NOS) profile. One patient carried *PTPN11* and *RUNX1* mutations with a normal karyotype (46,XY), while the other exhibited a *WT1* mutation with a translocation *t(8;21).* Blast infiltration was relatively low (35–50%), and both cases exhibited CD34+ and HLA-DR+ myeloid immunophenotypes (MPO+, CD13+, and CD33+). Aberrant immunophenotypic subpopulations were detected, with markers such as CD2+, CD7+, and CD19dim in the t(8;21) case and CD2+ and CD7+ in the other.

### 3.4. Complication Analysis

**Figure 9:**
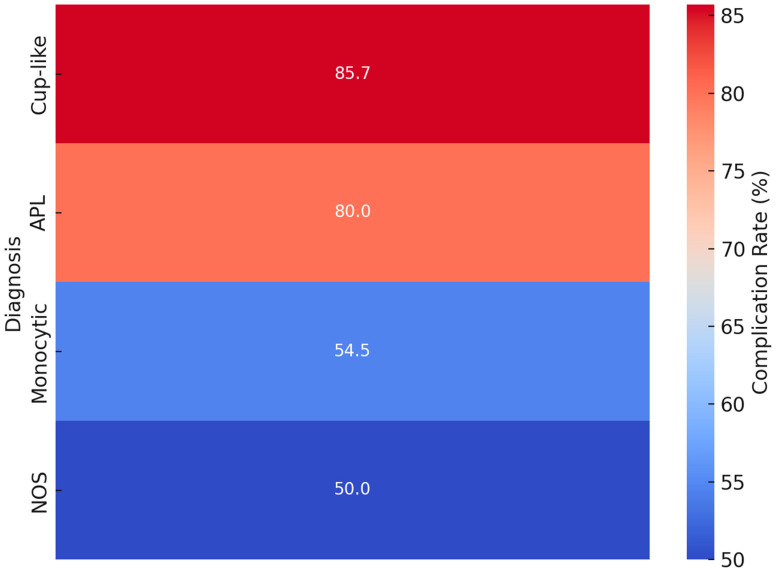
Complication rates across different morphological groups of acute myeloid leukemia (AML). The intensity of the color corresponds to the percentage of complications within each group, with the “cup-like” morphology showing the highest rate of complications.

We analyzed the complication rates in AML patients with overt DIC. DIC-related complications were defined as hemorrhagic or thromboembolic events. The analysis of complications across the different morphological groups revealed notable variations in the complication rates. Among patients with APL, 80% (four out of five patients) experienced complications, indicating a high prevalence of complications within this group. Similarly, patients with cup-like morphology exhibited the highest complication rate at 85.7%, with six out of seven patients affected. In contrast, the monocytic morphology group demonstrated a slightly lower complication rate of 54.5%, as 6 out of 11 patients experienced complications. Finally, the NOS group showed the lowest prevalence of complications, with only 50% of the patients affected. However, the NOS group comprises two patients, limiting the robustness of any conclusions derived from this subgroup. The data on morphological patterns and complications are presented in Figure 9. The overall complication rate across all morphological groups was 68%. The immunocytological analysis, covering the entire patient cohort including both APL and non-APL AML, reveals that CD34-/dim was very common, observed in 13 patients, while HLA-DR-/dim was also considerably frequent, identified in 8 patients. Therefore, among patients with complications, CD34-/dim was observed in 61.5%, while HLA-DR-/dim was present in 62.5%, highlighting the notable prevalence of these markers in this group. In the analysis of monocytic markers, CD4+ and CD14+ were more frequently associated with cases without complications, suggesting a potential link to a less severe clinical course. In contrast, CD64+ was predominantly observed in cases with complications, indicating a potential role in more severe or complex presentations. However, it is noteworthy that CD64 is also commonly expressed in myeloid leukemias, which could explain its presence in complicated cases due to its association with aggressive hematological conditions. The raw data of the analyses are available in the Supplementary Materials for review. While the current sample size does not allow for statistically significant differences to be demonstrated, certain trends can still be observed and may provide valuable insights.

## 4. Discussion

AML with overt DIC represents a formidable challenge, necessitating swift diagnostic and therapeutic interventions. APL, well known for its severe coagulopathy, demands immediate initiation of all-trans retinoic acid (ATRA), which not only targets leukemic cells, but also ameliorates coagulopathy [5,6]. In non-APL AML, alternative hemostatic therapies, such as recombinant human soluble thrombomodulin, have emerged as promising options for managing severe DIC [14,15].

Interestingly, our data reveal that while APL exhibits a more pronounced coagulopathy during the acute phase, the overall complication burden was higher in non-APL AML. This finding challenges the traditional view of APL as the archetype of DIC and highlights the need for vigilance across all AML subtypes. Moreover, patients with APL, although at high risk during the acute phase, tend to achieve sustained remissions once the critical period is managed. In contrast, non-APL AML presents a continuous risk profile, with persistent complications and higher overall mortality.

Our work underscores the pivotal role of hematopathological diagnostics in elucidating the interplay between leukemia and coagulopathy. The integration of NGS analyses revealed a notable enrichment of *WT1* mutations in our study cohort. Previous studies have largely focused on clinical manifestations of DIC [16,17], whereas this study places the diagnostic precision of hematopathology at the forefront. The association of *WT1* with DIC broadens the understanding of its pathogenic role in AML and warrants further investigation.

The frequent co-occurrence of *FLT3* and *NPM1* mutations in DIC-associated AML, particularly in cases with normal karyotypes, underscores their significance as drivers of leukemic progression and coagulopathy. These mutations exacerbate hypercoagulability through mechanisms such as cytokine overproduction and enhanced endothelial activation, contributing to a more aggressive disease course [18,19]. Notably, studies have highlighted their combined presence as a common mutational signature in high-risk AML, often linked to poorer outcomes and increased rates of DIC [16]. High prevalence rates of *DNMT3A* mutations in our cohort were notable, suggesting a potential link between epigenetic dysregulation and DIC in AML. Interestingly, other epigenetic regulators, such as *TET2*, were underrepresented, reinforcing the hypothesis of a more direct causative relationship between *DNMT3A* mutations and DIC in AML. Cytogenetic analyses revealed that normal karyotypes predominated in non-APL AML, shifting the focus from chromosomal aberrations to sequence-level mutations. This distinction highlights the importance of molecular diagnostics in identifying high-risk features in AML patients with unremarkable cytogenetic profiles. Nevertheless, we identified two cases with 11q23 rearrangements, a cytogenetic abnormality linked to severe coagulopathy in non-APL AML. These cases exhibited clinical features reminiscent of APL, including increased tissue factor expression and hyperfibrinolysis, which exacerbate bleeding and thrombotic risks [20].

Our findings reveal a high prevalence of CD34-negative/dim and HLA-DR-negative/dim blast populations in AML cases complicated by DIC. This immunophenotypic profile is consistent with a prior clinical study reporting a similarly high frequency of CD34-negative and HLA-DR-negative blast cells, underscoring its significance in the pathophysiology of AML-associated coagulopathy [17]. Further supporting this observation, experimental studies on myeloid differentiation have demonstrated that the attenuation of CD34 and HLA-DR expression is characteristic of the transition from myeloblasts to promyelocytes, a key step in the myeloid maturation process. This transition is mechanistically relevant to APL, where promyelocytes are the dominant leukemic cell type, often associated with severe coagulopathies due to increased procoagulant activity and hyperfibrinolysis. Together, these findings suggest that the immunophenotypic shifts observed in our study not only reflect advanced myeloid differentiation, but also indicate a potential link to the pathogenesis of DIC in AML, mirroring features commonly seen in APL [21,22].

This study also delineates three hematopathological patterns in AML complicated by DIC, offering a framework for precise diagnosis and tailored management. APL remains the most strongly associated with DIC and is characterized by severe coagulopathy, high *FLT3* burden, and distinct morphological features such as Auer rods and faggot bundles. The aggressive acute phase underscores the need for immediate ATRA therapy to stabilize patients and achieve remission [18,23]. AML with *FLT3* and/or *NPM1* mutations exhibited distinct characteristics, including a high prevalence of cup-like blasts and a high mutational burden. The aggressive nature of this subtype, combined with its distinct morphological and immunophenotypic features, underscores the importance of rapid diagnosis and intervention [19,23]. AML with monocytic differentiation displayed monoblastic morphology, *DNMT3A* mutations, and complex karyotypes, with a strong association with monocytic markers such as CD4, CD14, and CD64. These findings provide diagnostic clarity and emphasize the heterogeneity of DIC in AML. The identification of these patterns, supported by advanced genetic and morphological analyses, emphasizes the critical role of hematopathological expertise in the early recognition and management of high-risk AML subtypes.

## 5. Conclusions

This study reinforces the importance of hematopathological expertise in diagnosing and managing AML with overt DIC. By delineating three distinct patterns, we provide a structured framework that pathologists can apply in practice to enhance diagnostic accuracy and optimize patient outcomes. Future research should aim to refine these patterns further, incorporating advances in molecular diagnostics to deepen our understanding of AML’s complex biology and its interplay with coagulopathy.

## Figures and Tables

**Table 1:** DIC score according to the International Society on Thrombosis and Haemostasis (ISTH). A score of ≥4 points indicates overt DIC.

	0	1	2	3
platelets/µL	≥100	50–99	<50	
fibrinogen (mg/dL)	≥100	<100		
prothrombin time%	>70	40–70	<40	
D-dimer µg/mL	<3		3–7	>7

**Table 2:** Comparison of hematological parameters between patients with non-APL AML and APL. Parameters include leukocytes (L), hemoglobin (Hb), thrombocytes (T), prothrombin time (PT), fibrinogen (Fib), D-dimer, and DIC score.

		L/µL	Hb (g/dL)	T/µL	PT (%)	Fib (mg/dL)	D-Dimer (µg/mL)	DIC Score (≥4)
Reference Range		4.0–10.0	>12.0 (F), >13.6 (M)	150–400	70–130	180–350	<0.5	
non-APL AML	mean	46.00	8.72	45.25	68.75	360.65	52.82	5.25
20 patients	std	53.50	1.66	27.42	21.98	190.72	68.90	0.97
	med	16.45	8.35	39.50	68.00	398.50	17.08	5.50
APL 5 patients	mean	36.74	11.06	30.80	64.40	136.20	33.60	5.40
	std	45.83	2.29	16.15	15.98	28.05	25.97	0.89
	med	12.00	11.90	27.00	62.00	130.00	22.01	6.00
Comparison	*p*	0.61	0.08	0.15	0.62	0.09	0.72	0.76

**Table 3:** Summary of morphologic, immunophenotypic, and genetic markers in APL cases.

APL					
Diagnosis	NGS	Cytogenetics	Blast/Promyelocytes	Cytomorphology	Immuncytolology
APL	CALR	t(15;17)	82%	promyelocytes	CD34dim, HLA-DR dim
	FLT3			Auer rods	MPO+, CD13+, CD33+, CD117+
				Faggot bundles	CD4dim, CD15+, CD14dim, CD64+
					CD22dim
APL	FLT3	t(15;17)	78%	promyelocytes	CD34-, HLA-DR-
	WT1			Auer rods	MPO+,CD13dim, CD33++, CD117+
	WT1				CD15+, CD64+
	WT1				CD22dim
APL-V	FLT3	t(15;17)	92%	bi-nucleated	CD34dim, HLA-DRdim
				reniform	MPOdim, CD13dim, CD33+, CD117-
				hypogranulation	CD64+
				Auer rods	CD2+
				Faggot bundles	
APL	no mut	t(15;17)	85%	promyelocytes	not carried out
				Auer rods	punctio sicca like aspirate
				Faggot bundles	
APL-V	DNMT3A	t(15;17)	93%	bi-nucleated	CD34-, HLA-DR dim
	FLT3			reniform	MPO+,CD13dim, CD33+, CD117+
				hypogranulation	CD64+
				Auer rods	CD2+,CD19+

**Table 4:** Summary of cytogenetic findings and mutational profiles in AML patients with a distinct pattern with mandatory FLT3 and/or NPM1 mutations and cup-like blast dominance.

AML FLT3 with Cup-Like Blast Dominance					
Patients	NGS	Cytogenetics	Blast Count	Blastmorphology	Immuncytolology
1	DNMT3A	46,XX	95%	pseudopods	CD34+, HLA-DR+
	FLT3			cup-like	MPO+, CD13dim, CD33+, CD117+
	NPM1			hypogranulation	CD14+,CD64+
	FLT3				
2	DNMT3A	46,XX	86%	pseudopods	CD34-, HLA-DR+
	FLT3			cup-like	MPO+, CD13+, CD33+, CD117+
	NPM1			hypogranulation	
	FLT3				
3	FLT3	46,XX	84%	Auer rods	CD34+, HLA-DR+
	NPM1			pseudopods	MPO+, CD13+, CD33+, CD117+
	WT1			cup-like	CD2+
				hypogranulation	subpopulation: CD4+, CD14+,CD15+,CD64+
4	NPM1	46,XY	87%	cup-like	CD34-,HLA-DR-
	DNMT3A			hypogranulation	MPO+, CD13+, CD33+, CD117+
	IDH1				CD15+, CD64+
5	FLT3	t(8;21)	90%	Auer rods	CD34+, HLA-DR+
	RUNX1				MPO+, CD13+, CD33+, CD117+
	ASXL1				CD64+
					CD19+, CD79dim
6	NPM1	46,XY	96%	hypogranulation	CD34-, HLA-DR+
	IDH1				MPO+, CD13+, CD33+, CD117+
	PTPN11				CD64dim
	SRSF2				
7	DNMT3A	46,XX	74%	cup-like	34dim, HLA-DR+
	FLT3			hypogranulation	MPO+, CD13+, CD33+, CD117+
	NPM1			vacuoles	CD19+, CD10+
	WT1				
	IDH1				

**Table 5:** Hematopathological features of AML patients with monocytic differentiation and DIC complications.

Monocytic Differentiation						
Patients	NGS	Cytogenetics	Blast Count	Blastmorphology	Immuncytology	
1	DNMT3A	11q23	89%	monoblasts	CD34+, HLA-DR+	
				vaculisation	MPO+, CD13+, CD33+, CD117+	
				monolobulated, oval	CD4+,CD14+,CD64+	
				basophil cytoplasm margin	CD7+, CD56+	
2	DNMT3A	CK	94%	monoblasts	CD34+, HLA-DR+	
	TET2			vaculisation	MPO+, CD13dim, CD33+, CD117+	
	TP53			monolobulated, oval	CD4+, CD14+, CD15+, CD64+	
	TET2			basophil cytoplasm margin	CD56+	
3	FLT3	46,XX	84%	monoblasts		
	NPM1			vacuolisation	CD34-, HLA-DR+	
	DNMT3A			monolobulated, oval	MPO-, CD13+, CD33+, CD117-	
				basophil cytoplasm margin	CD4+, CD14+, CD15+, CD64+	
4	DNMT3A	CK	83%	monoblasts, pomonocytes	CD34-, HLA-DR-	
	TET2			vacuolisation	MPO-, CD13+, CD33+, CD117+	
	NPM1			monolobulated, convoluted	CD4+, CD14+, CD15+, CD64+	
	TP53					
5	TP53	no met.	43%	promonocytes	CD34-, HLA-DR+	
	TET2			vacuolisation	MPO+, CD13+, CD33+, CD117+	
				basophil cytoplasm margin	CD4dim, CD14-, CD15++, CD64+	
					CD56	
6	DNMT3A	46,XY	90%	monoblasts, pomonocytes	CD34+, HLA-DR+	
	FLT3			vacuolisation	MPOdim, CD13+, CD33+	
	NPM1			convoluted, oval	CD15++, CD64+	
7	DNMT3A	46,XX	88%	monoblasts, pomonocytes	CD34+, HLA-DR+	
	FLT3			vacuolisation	MPO+, CD13+, CD33+, CD117+	
	NPM1			monolobulated, convoluted	CD64++	
	3xWT1			basophil cytoplasm margin		
8	TET2	CK	92%	pseudopodes	CD34+, HLA-DR+	
	KRAS			monoblasts, pomonocytes	MPO+, CD13+, CD33+, CD117+	
				vacuolisation	CD4+, CD14+,CD15+, CD64+	
				monolobulated, convoluted	CD10+, CD56+	
				basophil cytoplasm margin		
				promoncytes		
9	DNMT3A	46,XY	44%	monoblasts, pomonocytes	CD34+, HLA-DR+	
	NPM1			vacuolisation	MPO+, CD13+, CD33+, CD117+	
	NRAS			monolobulated, oval	CD4+, CD14+, CD15+, CD64+	
				basophil cytoplasm margin		
10	FLT3	11q23	92%	monoblasts	CD34-, HLA-DR-	
				vacuolisation	MPO-, CD13-, CD33+, CD117+	
				monolobulated, oval	CD15+, CD64++	
				basophil cytoplasm margin	CD56dim	
11	NPM1	46,XX	56%	monoblasts, pomonocytes	CD34dim, HLA-DR-	
				vacuolisation	MPO+, CD13+, CD33+, CD117+	
				monolobulated, convoluted	CD4+, CD14+, CD15+	
				basophil cytoplasm margin		

## Data Availability

The original contributions presented in this study are included in the article/Supplementary Material. Further inquiries can be directed to the corresponding author.

## Supplementary Materials

The following supporting information can be downloaded at: https://www.mdpi.com/article/10.3390/diagnostics15030383/s1.
